# Identifying Knowledge Gaps among LVAD Candidates

**DOI:** 10.3390/jcm8040549

**Published:** 2019-04-23

**Authors:** Meredith Buchberg Trejo, Kristin M. Kostick, Jerry D. Estep, J.S. Blumenthal-Barby

**Affiliations:** 1 Center for Medical Ethics and Health Policy, Baylor College of Medicine, One Baylor Plaza, Suite 310D, Houston, TX 77030, USA; kristin.kostick@bcm.edu (K.M.K.); Jennifer.blumenthal-barby@bcm.edu (J.S.B.-B.); 2 Department of Cardiovascular Medicine, Cleveland Clinic, 9500 Euclid Ave. J3-4, Cleveland, OH 44195, USA; estepj@ccf.org

**Keywords:** ventricular assist device, mechanical circulatory support, heart failure, education, consent, knowledge

## Abstract

Education is an important aspect of evaluation and consent for left ventricular assist device (LVAD) candidates. A better understanding of candidate knowledge during the education process can help identify knowledge gaps and improve informed consent processes. This paper presents the results from a validated, LVAD-specific Knowledge Scale administered to candidates before and after education to identify items most and least frequently answered correctly. At baseline and 1-week, both candidates educated with a standard education and an LVAD-specific decision aid were most likely to answer logistical items relating to support and self-care correctly with ≥90% of candidates answering these items correctly after education. Candidates were least likely to answer questions about risks, transplant eligibility, and expenses correctly with <60% of candidates answering them correctly after education. Items with the greatest improvement in correct answers from baseline to 1-week were primarily related to the logistics of living with an LVAD. Candidates educated with the decision aid showed significant improvements on more knowledge items including those related to the forecasting of recovery and complications when compared to candidates educated with a standard education. The 20-item scale provides a standardized way for clinicians to identify knowledge gaps with LVAD candidates, potentially helping to tailor education. Targeted improvements in LVAD education should focus on the understanding of risk and potential complications to ensure that decision-making and informed consent processes emphasize both the patient and clinicians’ conceptualizations of knowledge needs for informed consent.

## 1. Introduction

Candidate education is an important aspect of the evaluation and consent processes for a left ventricular assist device (LVAD). Knowledge and understanding of current illness, treatment options, risks, benefits, and the requirements for device use and maintenance are cited as key domains in the psychosocial evaluation of candidates for long-term mechanical circulatory support in recent consensus-based recommendations supported by numerous professional societies including the International Society for Heart and Lung Transplantation [1]. Confirming that candidates are knowledgeable about these aspects helps verify their capacity to participate in the decision-making process. Furthermore, it enhances their ability to make decisions that align with their needs and values, thereby enhancing an environment of shared decision-making and patient-centered care [2]. Decision aids (DAs) may be a particularly useful tool to use during education as they have been shown to significantly increase patient knowledge when compared to standard care [3,4].

Although candidate education is a standard part of evaluation at most LVAD centers, variability exists in how it is delivered and how comprehension is evaluated. Available LVAD education materials include decision aids, brochures, websites, and videos developed by device companies, universities, and professional societies. Past evaluation of available LVAD education resources and informed consent documents has found substantial diversity in the readability, presentation of risks and benefits, and use of updated information [5,6]. Although LVAD centers may use institutionally-developed methods to assess candidate knowledge, many utilize informal methods such as the teach back method, particularly for items related to self-care and device management [7].

To our knowledge, only three standardized measures for evaluating LVAD candidate knowledge exist that reflect the breadth of information relayed during education including self-care, but also treatment options, lifestyle changes, benefits and risks [8,9,10]. The limitations of some of these measures include open-ended questions [9], which are difficult to consistently score, and specificity to destination therapy [10], which limits its clinical reach.

A past study found that a majority of patients were unable to answer items relating to survival, complications, transplant eligibility, and end of life issues correctly after consent and implantation [9]. However, little other empirical evidence exists regarding the specific knowledge LVAD candidates have both before and after receiving LVAD education [9,11,12]. Furthermore, it is unknown how candidate knowledge aligns with their informational preferences. While the use of DAs has been shown to significantly increase LVAD candidate knowledge [13,14], no information exists as to what specific types of knowledge it improves, and which knowledge gaps remain after education.

The primary aim of our study was to identify knowledge gaps that persist among LVAD candidates after using standard education or a validated LVAD decision aid designed to enhance patient-centered care. A better understanding of specific LVAD candidate knowledge as well as knowledge most likely to improve during education is necessary to identify knowledge gaps, tailor education, facilitate shared decision-making, and ensure providers elicit fully informed consent.

## 2. Methods

### 2.1. Knowledge Scale Development

A 20-item Knowledge Scale (KS) was used to assess candidate knowledge. The LVAD KS’s development and validation is described in detail elsewhere [8]. In summary, the LVAD KS was developed using inductive qualitative methods and deductive quantitative validation of scale items to determine content and face validity. The KS showed good internal consistency and reliability, and high acceptability during cognitive testing.

Interviews were conducted with existing LVAD candidates, individuals living with an LVAD, LVAD decliners, caregivers, and healthcare providers (i.e., cardiologist, cardiothoracic surgeon, LVAD coordinator) in order to ensure that the KS included items deemed important and necessary by all relevant stakeholders. Top-ranked informational needs reported by patients and candidates included mobility, lifestyle, technical device aspects, and self-care. Providers ranked emergency contact information, re-hospitalization risk, and recovery process as top informational needs.

Patient consultants remained engaged at every step of the development and validation process to ensure the overall focus and outcome was a patient-centered and patient-friendly tool. The scale covers key logistics associated with an LVAD including technical aspects of the device (i.e., overall purpose, understanding of battery alerts,), self-care (i.e., frequency of driveline cleaning, use of blood thinners, financial costs), use of and need for outside support systems (i.e., caregiving requirements, when to contact the LVAD team), and mobility (i.e., activities LVAD patients can do). The scale also included items designed to assess the candidate’s understanding of post-VAD recovery (i.e., total hospitalization time, estimated time in ICU), risks (i.e., risk of stroke, re-hospitalization, death, possible complications if the LVAD stops), future transplant (i.e., relationship between LVAD and transplant if bridge to transplant), and quality of life (i.e., potential improvements in overall well-being). Possible scores ranged from 0–100, with higher scores indicating higher knowledge levels. The full KS with response sets is available online at www.lvaddecisionaid.com.

### 2.2. KS Administration

**Figure 1:**
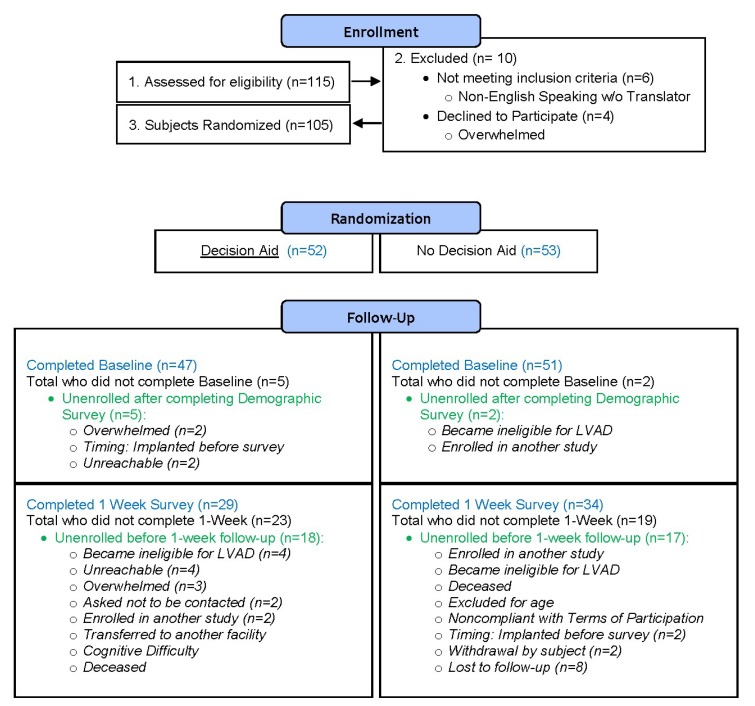
Participant flow chart for the LVAD Decision Aid Trial.

LVAD candidates participating in a trial to test the effectiveness of a LVAD DA were randomized after baseline to receive standard education or standard education plus the LVAD DA. No demographic or clinical characteristics were significantly different between the groups. Candidates completed the KS at baseline (pre-LVAD education) (*n* = 51 standard education group; *n* = 47 decision aid group) and 1-week after education (*n* = 34 standard education group; *n* = 29 decision aid group) [13]. Figure 1 shows the detailed enrollment, attrition, and data collection flow. Participants were recruited between July 2015 and January 2017 from five U.S. medical centers that reported a high volume of LVAD implantations. We report the findings at baseline and 1-week, as this represents the crucial decision-making period during which most candidates make the decision as to whether to accept LVAD treatment. 

### 2.3. Analysis

Item difficulty was calculated for each item in the KS in each education group using the number of individuals answering the item correctly out of the total number who participated at that time point. Improvement in correct responses for individual KS items was calculated using the change in item difficulty between baseline and 1-week completion of the KS. Chi-square and Fisher’s exact tests were used to test for significant changes from baseline to 1-week in the proportion of correct responses in each group. Previous work has shown that using the LVAD DA significantly increases overall candidate knowledge as compared to standard education [13]. The goal of this analysis is to identify specific knowledge gaps that remain after two different types of candidate education and the types of information that are the most and least likely to improve during the education process. This information will help provide clinicians using either type of education with a better understanding of the areas of education that may need increased tailoring and attention.

## 3. Results

LVAD candidates completing the KS at baseline were primarily male (77%) and Caucasian (68%). The average age of participants was 60 years (standard deviation (SD) = 12), with 41% designated as bridge to transplant and 57% as destination therapy. A full demographic profile of the participants has been previously reported in [13].

### 3.1. Candidate Knowledge

At the baseline, there were no significant differences in knowledge scores between candidates receiving the standard education and the candidates receiving the DA.

LVAD candidates exhibited high levels of knowledge at baseline and 1-week on certain logistical items relating to the need for a caregiver, the role of the LVAD clinical team, medication use, the purpose and function of an LVAD, supplies to carry outside the home, and types of possible complications. The items answered correctly by ≥90% of respondents in either group at 1-week are presented in Table 1.

At the baseline and 1-week, candidates least frequently correctly answered certain items relating to forecasting of re-hospitalization, complications if the LVAD stops, mortality rates, LVAD maintenance expenses, and the impact of an LVAD on possible transplant eligibility, with <60% of candidates in either group answering these items correctly at 1-week (Table 2).

### 3.2. Changes in Knowledge

At 1-week post-education, candidates receiving the LVAD DA scored significantly higher on the KS (mean = 67.8, SD = 15.6) compared to candidates receiving the standard education (mean = 59.3, SD = 12.4, *p* = 0.02). Changes in candidate knowledge for each KS item in each education group are shown in Table 3.

#### 3.2.1. Candidates Educated with Decision Aid

Candidates educated with the DA saw significant improvements (*p* < 0.05) in correct responses for eleven KS items including logistical and self-care items relating to care team support (#4), supplies to carry outside the home (#10), battery alerts (#12), need for caregivers (#15), driveline self-care (#16), use of blood thinners (#18), and for forecasting items related to potential complications (#3), re-hospitalization (#5), ICU recovery (#6), 2-year survival (#8), and factors associated with positive LVAD experiences (#20). Post-education, over 75% of candidates receiving the DA were able to correctly answer these items related to logistics and the forecasting item about potential complications. These candidates exhibited the smallest improvements in correct answers for forecasting items about factors affecting surgical recovery (#2), average hospital stay (#7), potential post-LVAD improvements (#14), transplant eligibility (#17), and what happens if the LVAD stops (#19). Post-education, these items were answered correctly by 34–62% of the candidates.

#### 3.2.2. Candidates Educated with Standard Education

Candidates receiving the standard education saw significant improvements on five items. Similar to candidates in the DA group, these candidates saw significant increases on certain logistical and self-care items relating to battery alerts (#12), driveline self-care (#16), and the use of blood thinners (#18). Candidates in this group also saw significant increases on certain items related to activities post-LVAD (#11) and potential post-LVAD improvements (#14) (Table 3). Post-education, these items were answered correctly by 56–97% of patients

The smallest improvements in correct answers were observed for forecasting items related to ICU stay (#6), total average hospital stay (#7), 2-year survival (#8), transplant eligibility (#17), and what happens if the LVAD stops (#19). With the exception of the item about ICU stay, these items were answered correctly by <60% of patients post-education.

The overall percentage of candidates answering each individual item correctly at 1-week was lower in the standard education group, with the exception of two items for which candidates in the standard education group answered the item correctly more frequently than candidates in the DA group: average hospital stay (DA = 62%; standard education = 71%) and potential post-LVAD improvements (DA = 48%; standard education = 56%).

One logistical item related to the purpose of an LVAD (#1) showed small improvement after education in both groups. However, this item was answered correctly by a majority of candidates at both baseline and 1-week post-education.

## 4. Discussion

Candidate education is a core aspect of the LVAD evaluation process. While ensuring that candidates are sufficiently informed is necessary for shared decision-making [2], it can be challenging in the LVAD setting where the amount and type of information can be overwhelming for candidates [15]. Results indicate that the patient-centered LVAD DA is effective at increasing overall candidate knowledge [13]. The results presented here revealed that candidates educated using the DA showed significant improvements on more KS items when compared to candidates receiving standard education. While both groups saw significant improvements on certain items relating to the logistics of self-care and technical aspects of the device, candidates educated with the DA also saw significant improvements on certain items relating to the forecasting of recovery and complications. 

However, regardless of education type, standard education, or LVAD DA, <60% of candidates were able to correctly answer forecasting items related to potential long-term complications or improvements including re-hospitalization, 2-year, mortality, what happens if the LVAD stops, quality of life, and transplant eligibility. These results are consistent with Edlund et al. [9] who found that LVAD candidate knowledge was lowest for similar items. Knowledge deficits relating to forecasting of surgical and post-surgical risks has been shown to be poor and widely variable across specialties including both underestimations and overestimations of risk, with some patients exhibiting a better understanding of potential benefits [16,17,18,19,20,21]. Patients may react defensively to information about risks and either sub-consciously avoid the information or spend minimal time considering it if they perceive it to be both highly threatening and highly relevant [22,23,24]. The items related to logistics and self-care, which saw significant improvements in correct responses in both groups, aligned with the information ranked as top informational needs among LVAD candidates [25], indicating that LVAD candidates may be more likely to retain knowledge about items they deem particularly salient prior to education, which may be attributed to availability bias [26]. While there is debate in the literature as to the precise amount and type of information necessary for patients to make high quality decisions [27], it is nevertheless important to note that the knowledge items cited above were specifically prioritized by clinicians in our sample as key informational needs for informed decision-making about LVAD (though they were not similarly ranked as top informational needs among the LVAD candidates in our sample) [25]. The difference between the LVAD candidates’ and clinicians’ desired knowledge highlights a question already posed in the literature on clinical decision-making, namely whether patients should be characterized as adequately informed when they feel satisfied with the information they have [28]. Our results suggest that LVAD clinicians would strongly disagree.

While candidates in our sample showed specific, persistent knowledge deficits post-education, it is possible that they felt informed enough to make a decision. Indeed, in our previous analysis, both groups reported high levels (>80%) of satisfaction with the decision-making process, indicating that they felt they received as much information as they wanted and received adequate information about the available treatment options [13]. However, when compared to those receiving standard education, those educated with the DA reported significantly greater satisfaction with life post-implantation and a larger percentage of individuals reporting that their LVAD outcomes were “very close to what I expected”. These results indicate that greater satisfaction post-implant may be linked to greater knowledge about the factors that are deemed important to both candidates and clinicians. Greater knowledge on both sets of items may afford candidates a greater ability to gauge what life (and LVAD-related challenges) will be like post-implant. This is supported by the fact that while quality of life is most often substantially improved by LVAD, it is also intimately linked to post-surgery complications and frequency of re-hospitalizations [29,30,31,32]. Our results showed that accurate forecasting about these particular aspects was improved by educating candidates using our DA, which presented risk information according to criteria specified by the International Patient Decision Aid Standards Collaboration [33].

Although providers may feel more comfortable discussing technical aspects of the device and surgical procedures [12], compared to complications and longer term survival numbers, providers should give extra attention to the way in which risk information is communicated to patients. Risk information may be better retained by patients if it is presented multiple times [34] or personalized [35]. Providers should also recognize that many DAs including the one described in this article, are not standalone tools and must be used in an interactive way with the patient to educate them, elucidate decision-making preferences, and identify goals and values that matter most to the patient. Giving a patient a decision aid with no discussion does not constitute shared decision-making and it is unlikely that risk information will “sink in”. Tools such as the KS offer a practical means for providers to systematically understand where knowledge gaps remain and ensure they are addressed during the course of shared decision-making.

### Limitations

Although candidates were recruited from five healthcare centers performing a high volume of LVAD implants, results may not be generalizable to all LVAD candidates and centers. In addition, one KS item (#13) was incorrectly scored for some candidates and we have omitted all responses for this question from our analysis. While 95% of candidates receiving the LVAD DA reported reading the entire DA [13], we have limited information about how providers in either group discussed specific types of information with candidates in practice. However, providers received training in the use of the DA with patients. We are currently working to evaluate contextual factors influencing real-world dissemination of the LVAD DA and fidelity of use including the communication of risks, in order to develop guidelines and training for how to most effectively use the full DA in clinical practice.

An additional potential limitation is that pre- and post-education knowledge gaps may differ with the use of a different LVAD knowledge scale. To date, no formal comparison has been made between existing, validated LVAD knowledge scales, of which there are at least two (see also [14]). However, similar overall knowledge scores post-education were observed in independent trials of each scale, indicating that the persistence of knowledge gaps may be a widespread issue. 

## 5. Conclusions

Clinicians offering LVAD therapy have a responsibility to ensure that candidates for LVAD receive the information that they and their clinicians consider to be important for informed decision making. Candidate education using a validated DA for LVAD led to improvements in patient desired knowledge about what to expect during recovery and how to master the logistics of self-care and technical aspects of the device. However, further attention needs to be paid to communicating very important knowledge about post-surgery risks, long-term complications and mortality, quality of life, and transplant eligibility where candidates showed continued knowledge gaps even after receiving a trial-tested form of LVAD education.

## Figures and Tables

**Table 1:** LVAD knowledge items answered correct most frequently across the baseline and 1-week.

Question	% Correct at Baseline: Standard Education Group (*n* = 51)	% Correct at Baseline: Decision Aid Group (*n* = 47)	% Correct at 1-Week: Standard Education Group (*n* = 34)	% Correct at 1-Week: Decision Aid Group (*n* = 29)
1. The LVAD is a device that…	80%	79%	88%	90%
3. Which of the following is NOT a potential LVAD complication?	61%	64%	74%	93%
4. When should you call the LVAD team for support?	84%	81%	97%	100%
10. When you leave your house for a day trip, you should always have with you…	39%	40%	56%	93%
15. In the first weeks after going home from surgery, LVAD patients need daily help from a caregiver. (T/F)	88%	83%	100%	100%
18. Why do you have to take blood thinners (such as Coumadin) after you get an LVAD?	78%	74%	97%	97%

**Table 2:** LVAD knowledge items answered correct least frequently across the baseline and 1-week.

Question	% Correct at Baseline: Standard Education Group (*n* = 51)	% Correct at Baseline: Decision Aid Group (*n* = 47)	% Correct at 1-Week: Standard Education Group (*n* = 34)	% Correct at 1-Week: Decision Aid Group (*n* = 29)
5. Which statement below most accurately reflects how likely it is that a patient will have to go back into the hospital within one year after getting an LVAD?	20%	17%	32%	55%
8. What percentage of patients are still alive 2 years after receiving an LVAD?	22%	17%	29%	41%
9. For most people, who covers expenses for additional LVAD maintenance supplies?	25%	30%	41%	48%
14. After getting an LVAD, most people experience improvements in which of the following?	33%	45%	56%	48%
17. How does an LVAD affect your future eligibility for a heart transplant, if at all?	25%	36%	35%	45%
19. What will happen to you if the LVAD stops?	18%	19%	24%	34%

**Table 3:** Changes in LVAD knowledge between pre- and 1-week post-education.

Increase in Correct Responses (Standard Education)	LVAD Knowledge Questionnaire	Increase in Correct Responses (Decision Aid Education)
8%	1. The LVAD is a device that…	11%
13%	2. Factors that affect how fast you recover after LVAD surgery include…	10%
13%	3. Which of the following is NOT a potential LVAD complication?	30% *
13%	4. When should you call the LVAD team for support?	19% *
13%	5. Which statement below most accurately reflects how likely it is that a patient will have to go back into the hospital within one year after getting an LVAD?	38% **
10%	6. How long should the average patient expect to stay in intensive care (ICU) after the operation?	40% **
10%	7. For the average patient, about how long is the total expected hospital stay (including intensive care and rehabilitation) after surgery?	9%
8%	8. What percentage of patients are still alive 2 years after receiving an LVAD?	24% *
16%	9. For most people, expenses for additional LVAD maintenance supplies (such as extra gauze, gloves and cleaning supplies, etc.) are covered by:	18%
17%	10. When you leave your house for a day trip, you should always have with you…	53% **
33% *	11. Which one of the following activities will be true after you receive an LVAD?	19%
26% *	12. When my LVAD battery power is getting low, the controller will alert me by:	37% *
3% ^†^	13. How likely is it that a patient with end-stage heart failure will be alive in one year, if he or she does not receive an LVAD?	−9% ^†^
23% *	14. After getting an LVAD, most people experience improvements in which of the following?	4%
12%	15. In the first weeks after going home from surgery, LVAD patients need daily help from a caregiver. (T/F)	17% *
38% **	16. How often will you need to clean your driveline?	42% **
10%	17. How does an LVAD affect your future eligibility for a heart transplant, if at all?	9%
19% *	18. Why do you have to take blood thinners (such as Coumadin) after you get an LVAD?	22% *
6%	19. What will happen to you if the LVAD stops?	15%
21%	20. Which one of the following does NOT provide an advantage for how well patients do with an LVAD?	36% **

* *p* < 0.05; ** *p* < 0.001; ^†^ Item incorrectly scored and not included in the current analysis. See Limitations Section.
